# Dysphagia Management and Cervical Auscultation: Reliability and Validity Against FEES

**DOI:** 10.1007/s00455-022-10468-8

**Published:** 2022-07-15

**Authors:** Mariam Jaghbeer, Anna-Liisa Sutt, Liza Bergström

**Affiliations:** 1 Institute of Neuroscience and Physiology, Department of Health and Rehabilitation, Speech and Language Pathology Unit, Sahlgrenska Academy at the University of Gothenburg, Gothenburg, Sweden; 2 Alian Al Aajalain Street, Amman, Jordan; 3 Critical Care Research Group, The Prince Charles Hospital, Adult Intensive Care Services, Brisbane, Australia; 4 Faculty of Medicine, University of Queensland, Brisbane, Australia; 5 Remeo Stockholm, Torsten Levenstamsväg 8, Stockholm, Sweden; 6 Division of Neurology, Department of Clinical Sciences, Karolinska Institute, Danderyd University Hospital, Stockholm, Sweden

**Keywords:** Validity, Reliability, Swallow, Respiratory, Deglutition

## Abstract

This study investigated the reliability and validity (sensitivity and specificity) of cervical auscultation (CA) using both swallow and pre-post swallow-respiratory sounds, as compared with Flexible Endoscopic Evaluation of Swallowing (FEES). With 103 swallow-respiratory sequences from 23 heterogenic patients, these swallows sounds were rated by eight CA-trained Speech-Language Pathologists (SLPs) to investigate: (1) if the swallow was safe (primary outcome); (2) patient dysphagia status; (3) the influence of liquid viscosity on CA accuracy (secondary outcomes). Primary outcome data showed high CA sensitivity (85.4%), and specificity (80.3%) with all consistencies for the *safe* measurement, with CA predictive values of $>$ 90% to accurately detect unsafe swallows. Intra-rater reliability was good (Kappa $=$ 0.65), inter rater reliability moderate (Kappa $=$ 0.58). Secondary outcome measures showed high sensitivity (80.1%) to identify if a patient was dysphagic, low specificity (22.9%), and moderate correlation *(r*_s_ $=$ 0.62) with FEES. A difference across bolus viscosities identified that CA sensitivities (90.1%) and specificities ($>$ 84.7%) for thin liquids were greater than for thick liquids (71.0–77.4% sensitivities, 74.0–81.3% specificities). Results demonstrate high validity and moderate-good reliability of CA-trained SLPs to determine swallow safety when compared with FEES. Data support the use of CA as an adjunct to the clinical swallow examination. CA should include pre-post respiratory sounds and requires specific training. *Clinical implications*: The authors advocate for holistic dysphagia management including instrumental assessment and ongoing CSE/review $+$ CA. Adding CA to the CSE/review does not replace instrumental assessment, nor should CA be used as a stand-alone tool.

**Supplementary Information:**

The online version contains supplementary material available at 10.1007/s00455-022-10468-8.

## Introduction

Dysphagia, if not optimally managed, negatively affects patient outcomes, hospital lengths-of-stay, medical and total health care costs [1–5]. For the individual, the burden of dysphagia is well documented, impacting a person’s nutrition and hydration status [6], quality of life and psychosocial status [7]. The importance of dysphagia identification and management cannot be underestimated. Research data have identified that early screening, assessment and management of dysphagia improve patient outcomes, lower the risk of pneumonia, and health-care costs [3].

Assessment of swallow function commonly occurs via (1) a Clinical Swallow Examination (CSE) and/or (2) instrumental assessments such as Fiberoptic Endoscopic Evaluation of Swallowing (FEES) and/or Videofluoroscopic Swallow Study (VFSS), the latter two considered to be the gold standard in dysphagia assessment [4, 5, 8]. In recent years, holistic management using evidence-based medicine principles have identified that all three assessments contribute to optimal dysphagia and oral intake management considering person-centered care [6, 8–10].

Evidenced-based medicine (EBM), first coined by Sackett [11], challenges clinicians to consider (1) relevant scientific evidence, (2) clinical judgement, with (3) patient values and preferences. Optimal dysphagia and oral intake management requires information not from one assessment only, but from all clinical signs, symptoms, patient factors, health support services and various influencing factors [8, 10, 12]. In recent years, the concept of EBM has expanded and now requires the clinician to consider also the health system (e.g. availability of services, treatments, assistive devices, health insurance coverage), and the service organization (e.g. spectrum and training level of health professionals, availability of diagnostic, and treatment devices) [13].

In terms of contemporary EBM principles, the management of safe and efficient swallowing along with adequate hydration and nutritional intake across all meal-times is a challenge often requiring high level skills and interpretation of instrumental assessments as well as ongoing clinical examination/reviews [8, 10, 14]. Instrumental assessments and clinical swallow examinations provide different yet complementary clinical information, and together they allow for improved person-centered care and EB dysphagia management, all in-line with the World Health Organization’s International Classification of Functioning, Disability and Health (ICF) model [8, 12–15]. For example, in terms of ICF it is essential to consider (1) body structures and functions (optimally assessed via instrumental exam), also (2) a person’s activity, participation, and (3) environmental and personal factors (information obtained from a range of sources, including ongoing dysphagia review and/or mealtime observation using the clinical swallow exam). To this end, the CSE is referred to as the CSE/review, since it is often utilized not only as an initial exam but also for ongoing dysphagia management and follow-up reviews [5, 6, 9, 12].

It is well-established that instrumental assessments are necessary for (1) diagnostics, (2) to identify the pathophysiological causes for dysphagia which subsequently underpins effective rehabilitation, and (3) trial compensatory and/or rehabilitation interventions [5, 6, 14, 16]. The CSE/review complements instrumental assessment. In line with dysphagia management as per ICF, the CSE/review allows for (1) at times, earlier assessment prior to an instrumental; (2) more regular re-assessment for identification of changes in dysphagia/oral intake abilities, depending on fluctuations in cognition, function and medical status, albeit acute or non-acute fluctuations; (3) the opportunity to review dysphagia, oral intake and feeding/compensatory maneuvers/strategies during an entire meal; (4) incorporate and adapt management to patient preferences/abilities; and (5) provide ongoing follow-up of patient/dysphagia progression to further adjust/upgrade diets and/or feeding strategies in a timely manner [5, 8, 9, 14].

In recent years, there has been a growing body of evidence, in both pediatric and adult research, identifying that using cervical auscultation as an adjunct to the CSE improves the accuracy of the CSE, as compared with instrumental assessment [17–19]. Cervical auscultation involves listening to and interpreting swallow sounds and swallow-respiratory coordination using stethoscopes, microphones or accelerometers [17–20]. Cervical auscultation, however, is considered controversial in some dysphagia circles with a 2016 systematic review identifying only six articles of moderate-good quality. Results reported sensitivity ranges from 23 to 94% and specificity 50 to 74% with poor to fair inter rater reliability [21]. This systematic review, however, excluded studies using an accelerometer or microphone, the latter predominantly used in pediatric cervical auscultation [17, 18, 22].

Recent work by Bergström & Cichero [12] identified that early CA studies, often reported poorer CA validity and reliability results [23–26]. These CA studies were conducted with suboptimal methodology, including (1) poor swallow-respiration sound and sampling methodology, (2) inconsistent/no CA training, (3) variable assessment parameters/ratings for CA versus instrumental assessment, and (4) no provision of definitions $-$ all critical factors for robust research within CA. Research that has focused upon the swallow-respiratory coordination and characteristics, indicates that CA is able to distinguish normal from abnormal swallows [18, 22, 27, 28]. Within CA research and non-CA dysphagia research, dysphagic swallows may be characterized by an incoordinated (inhalation post swallow) or changed pre-post swallow-respiratory pattern, longer deglutition apnea, longer swallow sounds and/or increased number of swallows per bolus [20, 29–31].

Given the incongruent literature and earlier poor CA methodology, the current study aimed to address these previous research limitations. A crucial component to swallowing, and therefore to CA, is the swallow-respiratory coordination. The physiology and swallow-respiratory interplay within both normal and dysphagic swallows is well described in the literature [22, 27, 28, 32–36]. Respiratory swallow patterns are represented by expiration-swallow-expiration as the most common pattern followed by inspiration-swallow-expiration as the second most frequent pattern [32, 33, 37]. Both of these patterns include a period of apnea (1) preceding the swallow response and (2) present during the swallow. Studies show that patients with dysphagia have disrupted swallow-respiratory coordination usually represented by an inspiration post swallow, which increases the risk of penetration or aspiration, and a shorter duration of post swallow expiratory sounds than compared with healthy swallow-respiratory sounds [29, 30]. Swallow-respiratory patterns which deviate from normal are prevalent in several different (adult) patient groups including stroke, chronic obstructive pulmonary disease/respiratory diseases, Parkinson’s disease, head and neck cancer [34–36, 38]. Within pediatrics (neonatal and older children), the (suck-) swallow-breathe synchrony is again essential for safe and efficient feeding and swallowing [8, 17, 18, 22, 31].

Although earlier CA research has not always included the swallow-respiratory component within their study samples [23–26], a growing body of research highlights this to be a critical feature for improved reliability and validity when compared against instrumental assessment [17, 19, 27, 28, 30]. Subsequently, this study aimed to address previous CA research limitations by ensuring not only (1) CA recordings of pre-post swallow respirations, but also (2) appropriate CA training, with (3) a structured rating protocol and established CA rating definitions. Specifically, the following aims were investigated.

1. Primary aim: To investigate the validity and reliability of CA-trained clinicians to identify if a swallow (including pre-post respiration) is safe on a particular consistency, as compared with a simultaneously recorded FEES reference test.
2. Secondary aims:
3. To investigate the validity and reliability of CA-trained clinicians to identify if a patient is dysphagic (as compared with simultaneous FEES) by listening to recorded swallow-respiratory sounds of a patient swallowing several thin and thick liquid boluses.
4. To investigate the effect of bolus viscosity on validity and reliability of CA-trained clinicians in identifying if a swallow is safe as compared with simultaneously recorded FEES.

## Methodology

This cross-sectional study was approved by the Swedish Ethical Review Authority, Dnr 1141–16 and conducted in accordance with Standards for Reporting Diagnostic Accuracy (STARD) [39].

### Swallow-Respiratory Sounds

The 103 swallow-respiratory sounds (of which 18 were duplicates, 20%) were prospectively recorded from 23 heterogenic patients that were referred for a FEES assessment over an 18-month period at Norra Älvsborgs Länssjukhus (NÄL) Hospital, Trollhattan, Sweden. (For patient characteristics, see Online Resource 1). Research data were collected for all consenting FEES patients (consecutive, convenience sampling). Swallow-respiratory sounds were recorded simultaneously during FEES using a Littmann E-3200 (electronic, AB Henry Eriksson, Sweden) stethoscope. The sample size of 23 patients was calculated to provide a power of 93.7% [40] based on the dichotomous value (dysphagia) and a population estimate of 40% [4] with a *p*-value of 0.05.

### Flexible Endoscopic Evaluation of Swallowing

A FEES procedure was chosen as the gold standard reference test to which CA ratings would be compared, as per literature [6, 8, 16]. A standardized FEES (based on Langmore’s protocol, 1988 [41]) was conducted by SLPs (*n* = 2), or Otolaryngologists (*n* = 4) with an accompanying SLP, with a range of FEES experience of 2–12 years. All FEES assessors had access to all patient background and clinical information which was reviewed prior to FEES. Food and liquid consistencies as per the International Dysphagia Diet Standardization Initiative (IDDSI) were presented to the patient (IDDSI-0 thin liquid, IDDSI-2 mildly thick liquids, IDDSI-4 extremely thick liquids, IDDSI-7 regular diet, biscuit), with a minimum of two boluses/consistency presented if no significant aspiration occurred, (see Online Resource 2 for FEES protocol). Immediately after reviewing the FEES images, the clinical researcher and endoscopist answered a set of clinical questions that included; (1) Is the swallow safe on this consistency? (2) What is the penetration-aspiration score? (3) Does the patient have dysphagia and what is the patient’s dysphagia severity as per Australian Therapy Outcome Measures (AusTOMs) [42]. These three clinical questions were discussed and determined via consensus rating post FEES. Consensus was reached 100% of the time.

The clinical questions regarding (1) swallow safety, (2) penetration-aspiration score, and (3) dysphagia rating, were chosen as outcome measures to reflect contemporary dysphagia management where evaluation of (1) swallow safety and (2) swallow efficiency is recommended for clinical and instrumental swallow assessments [4, 8]. Instrumentally, swallow safety is often measured using the penetration-aspiration scale [43] and has also been defined as a patient’s capacity to swallow required food and fluids without respiratory complications occurring [4]. The secondary outcome measure for this study, that of ‘dysphagia rating’, was chosen since (a) this is a core aspect of dysphagia research [16], (b) this rating incorporates all symptoms of dysphagia, including (c) instances where patients may be considered safe on a specific consistency yet still present with poor swallow efficiency and/or other dysphagia characteristics [1, 19]. Dysphagia was defined with ratings based on AusTOMs [42].

### Cervical Auscultation (Swallow-Respiratory) Recordings

Swallow-respiratory sounds were recorded via a Littmann E-3200 (electronic) stethoscope placed by the CA clinical researcher (SLP, 19 years clinical CA experience and 6 years CA research experience) on the lateral border of the trachea inferior to the cricoid cartilage, as per previous research [44]. A total of 20–30 seconds of swallow-respiratory-sound recordings were taken simultaneously during FEES with the following stethoscope settings: Position = supine; Filter = diaphragm mode (20 – 2000 Hz); Sound Amplification = level 2. Swallow-respiratory sound recordings included the following: (a) two to three inhalation-exhalation sequences, (b) swallow sounds, (c) two to three inhalation-exhalation sequences – recorded simultaneously during the FEES. When the patient required more than one swallow to complete the oral-pharyngeal deglutition phase, then all swallow-respiratory sounds were recorded where possible. Some swallow-respiratory sound recordings were edited to remove extraneous noise and sounds, such as stethoscope rubbing or patient voicing, resulting in some swallow-respiration sounds being $<$ 30 seconds.

### Cervical Auscultation Rating

Eight SLPs from Australia, Sweden and UK were recruited via national dysphagia networks, email lists, and online platforms. Inclusion criteria consisted of (1) a minimum of two years’ experience in dysphagia management, (2) previous attendance at a (minimum) one-day workshop for CA, and (3) currently using CA as part of their routine dysphagia management on a daily-weekly basis.

A one-hour consensus training was developed by the authors, based on previous research [12, 18–20, 27–30] and sent to all raters who were required to rate if the swallow-respiratory sequences were safe and/or dysphagic. See Online Resource 3 as an example of consensus training information regarding swallow-respiratory sound interpretation. The consensus training included (a) descriptions of swallow-respiratory sounds, and (b) 12 sound clips of normal and dysphagic swallow-respiratory sequences with different food/liquid consistencies. These sound clips included; normal swallow-respiratory sounds (two 5 ml IDDSI-0, two 10 ml IDDSI-0, one 10 ml IDDSI-2, one 10 ml IDDSI-3) and dysphagic swallow-respiratory sounds (two 10 ml IDDSI-0, two 10 ml IDDSI-2, two 10 ml IDDSI-4). These swallow-respiratory training samples were independent to the actual study sample sent to raters.

Raters then received a total of 28 patient files (23 patients plus five duplicates, 20%), resulting in 103 swallow-respiratory sounds in a randomized patient order. For this study, only IDDSI-0, IDDSI-2 and IDDSI-4 were rated by CA-trained clinicians, however not all 23 patients had a full complement of IDDSI-0, IDDSI-2 and IDDSI-4 swallow recordings due to swallow safety concerns, patient voicing during the assessment or other edits, as mentioned above. (Number of IDDSI-0, 2 and 4 swallows presented to the CA raters are reported under Results section.) These swallow-respiratory files and a CA Rating Form were sent to SLP raters as a WeTransfer link [45] via email. The CA rating form included the same three clinical questions as per FEES, (1) Is the swallow safe on this consistency? (2) Does the patient have dysphagia? (3) What is the dysphagia severity for the patient, based on AusTOMs [42] – see Online Resource 4. Raters were given set instructions and advised to rate in one-hour blocks, using noise canceling headphones or in-ear-piece with a good fit, in a quiet room. Raters were blinded to patient information, including demographics, medical history, diagnosis and FEES ratings. Two swallow-respiratory sounds were excluded from the study because they were not rated by one of the raters. No adverse events were reported during the FEES or CA ratings.

### Methodological Validity

This study was conducted according to STARD with several methodological considerations, including content validity, criterion validity, reliability and feasibility [9, 39]. The population (23 heterogenic patients with variable dysphagia characteristics) is reflective of the usual dysphagia caseload seen at a large regional hospital in Sweden, presenting a relevant study population for content validity. The outcome measures (if the swallow was safe/dysphagic) were chosen considering content validity (relevant measurement items) since patients may be considered safe on a specific consistency yet still present with poor efficiency and/or dysphagia characteristics [1, 19] as per instrumental or clinical assessment. Furthermore, the same patient may be unsafe on one consistency yet safely swallow another consistency without signs of penetration/aspiration [19]. For criterion validity, diagnostic performance, and reliability, see statistical analysis and results below.

## Statistical Analysis

IBM® SPSS® Statistics platform was used for the statistical analyses. For the primary outcome (binary) *safe* measurement, CA validity was calculated using sensitivity and specificity, and positive and negative predictive values. Area under the Receiving Operating Curve (aROC) and Confidence Intervals (95%) were also calculated. Cohen’s Kappa was used to determine intra rater reliability, Fleiss’ Kappa (Kf) for inter rater reliability. For binary *dysphagic* measurement sensitivity and specificity were measured, Gwet’s AC1 was used for intra rater reliability instead of Cohen’s Kappa due to Kappa paradox [46]. Fleiss’ Kappa was used for inter rater reliability. Lastly, for ordinal *severity* measurement, Spearman’s correlation coefficient (*r*_s_) calculated the CA ratings correlation with the FEES assessment, weighted Kappa (Kw) was used to measure CA intra rater reliability, and Mean of all weighted Kappas was used for inter rater reliability. Interpretation of statistical analyses was in accordance to cut-off points as per Table 1.

**Table 1:** Statistical analyses interpretation

Sensitivity and specificity	0.90–100	0.75–0.89	0.60–0.74	0.40–0.59	0.10–0.24
	Very high values	High values	Moderate-high values	Moderate values	Low values
Correlation with FEES	0.90–1.00	0.70–0.89	0.40–0.69	0.10–0.39	0.00–0.10
	Very strong correlation	Strong correlation	Moderate correlation	Weak correlation	Negligible correlation
Agreement using Kappa statistic	Kappa 0.81–1.00	Kappa 0.61–0.80	Kappa 0.41–0.60	Kappa 0.21–0.40	Kappa < 0.20
	Very good agreement	Good agreement	Moderate agreement	Fair agreement	Poor agreement

Lange and Lippa [47], Schober and Schwarte [48], Altman [49]

## Results

The 103 swallow-respiratory sounds (of which 18 were duplicates, 20%) were collated from 23 patients with a mean age of 68 years (range: 34–83). Patients were heterogenic in both dysphagia presentation/severity (dysphagic = 17, non-dysphagic = 6) and medical diagnosis (see Online Resource 1). Of the patients with dysphagia (74% of the cohort), 6/17 presented with mild dysphagia, 7/17 with moderate, and 4/17 with moderate-severe dysphagia, as per FEES. Of the 85 original swallows, 61 were safe (72%) and 24 were unsafe (28%). Unsafe swallows included: thin liquids = 16/39 (41%), mildly thick liquids = 4/23 (17%), extremely thick liquids = 4/23 (17%).

### Primary Outcome Measure: Is the Swallow Safe?

The validity (sensitivity and specificity) of CA as compared with FEES data was high—for all consistencies (IDDSI-0, IDDSI-2, IDDSI-4) combined (sensitivity = 85.4%, specificity = 80.3%) with highest validity results (sensitivity = 91.1%, specificity = 84.7%) for thin liquids (IDDSI-0). The negative predictive values (> 90%) identified the high accuracy of CA to detect patients who were truly not safe, as per FEES. See Table 2 for further validity and reliability results.

**Table 2:** Primary outcome measure: CA validity and reliability across swallow-respiratory sounds for the ‘safe’ measure

	Is the swallow safe?					
	IDDSI-0 (5 ml) *n* = 160	IDDSI-0 (10 ml) *n* = 159	IDDSI-0 (5 ml & 10 ml) *n* = 319	IDDSI-2 (10 ml) *n* = 183	IDDSI-4 (10 ml) *n* = 181	All consistencies *n* = 683
Statistical analyses						
Sensitivity	93.7%	88.3%	91.1%	71%	77.4%	85.4%
Specificity	83.7%	86.0%	84.7%	74%	81.3%	80.3%
aROC	0.89 (CI = 0.83–0.94)	0.87 (CI = 0.81–0.94)	0.88 (CI = 0.84–0.92)	0.73 (CI = 0.62–0.83)	0.79 (CI = 0.70–0.89)	
Intra rater exact agreement	88%	79%	84%	90%	75%	83%
Intra rater reliability	K = 0.63	K = 0.41	K = 0.54	K = 0.79	K = 0.45	K = 0.65
Inter rater exact agreement	55%	58%	56%	43%	52%	52%
Inter rater reliability	Kf = 0.70 (CI = 0.62–0.79)	Kf = 0.66 (CI = 0.58–0.75)	Kf = 0.69 (CI = 0.63–0.75)	Kf = 0.42 (CI = 0.34–0.50)	Kf = 0.50 (CI = 0.41–0.57)	Kf = 0.58 (CI = 0.54–0.62)
PPV	79.7%	81.5%	80.6%	36.7%	52.9%	63.2%
NPV	95.1%	91.4%	93.2%	92.3%	94.4%	93.3%
FP (type I error)	16.3%	14%	15.3%	26%	18.8%	19.7%
FN (type II error)	6.3%	8.6%	8.9%	29%	22.6%	14.6%
Exact agreement	88%	88%	86%	74%	70%	77%

*n* = *number of swallows*, aROC = area under the Receiver-Operating Curve, CI = Confidence Interval (95%), K = Cohen’s kappa, Kf = Fleiss’ kappa, PPV = Positive Predictive Value, NPP = Negative Predictive Value, FP = False Positive, FN = False Negative

Intra rater reliability for all consistencies combined was good (83% agreement, Kappa = 0.65), with highest agreement for mildly thick liquids (IDDSI-2)—90% agreement and Kappa = 0.79. Inter rater reliability in detecting if the swallow was safe, for all consistencies combined, was moderate (Kappa = 0.58), and highest for thin liquids (IDDSI-0) with a Kappa of 0.69. See Table 2.

### Secondary Outcome Measures: Does the Patient Have Dysphagia?

Sensitivity for CA to detect patients with dysphagia was high (80.1%), specificity low (22.9%). Intra rater reliability was very good (Gwet’s AC1 = 0.9) and inter rater reliability fair (Kappa = 0.30). See Table 3 for summarized results.

**Table 3:** Secondary outcome measures: summary of CA validity and reliability across swallow-respiratory sounds for the ‘dysphagic’ and ‘severity’ measures

	Does the patient have dysphagia? n = 23	What is the patient’s dysphagia severity? n = 23
Statistical analyses		
Correlation with FEES		*r*_s_ = 0.62
Sensitivity	80.1%	
Specificity	22.9%	
aROC	0.52 (CI = 0.42–0.61)	
Intra rater exact agreement	95%	48%
Intra rater close agreement		88%
Intra rater reliability	Gwet’s AC1 = 0.9	Kw = 0.49 (CI = 0.33–0.65)
Inter rater reliability	Kf = 0.30 (CI = 0.22–0.38)	Kw = 0.41^a^ (CI = 0.16–0.64)^b^
PPV	74.7%	
NPV	28.9%	
FP (type I error)	77.1%	
FN (type II error)	19.9%	
Exact agreement	65%	27%
Close agreement		81%

*n* = *Number of patients*, *r*_s_ = Spearman’s Correlation Coefficient, aROC = Area Under the Receiver-Operating Curve, CI = Confidence Interval (95%), Kw = Weighted Kappa, ^a^ = mean of all 28 Weighted Kappas, ^b^ = mean of all low limits and upper limits, Kf = Fleiss’ kappa, PPV = Positive Predictive Value, NPP = Negative Predictive Value, FP = False Positive, FN = False Negative

### What Is the Patient’s Dysphagia Severity Rating?

CA ratings correlation with FEES regarding patient dysphagia severity (ordinal scale) was moderate (Spearman’s Correlation Coefficient = 0.62). Intra and inter rater reliability were moderate (Kappa = 0.49, mean of Kappa = 0.41). See Table 3 for summarized results.

## Discussion

In this heterogenic dysphagia population, high CA validity (sensitivity and specificity > 80%) for detecting safe swallows with all consistencies was demonstrated, with > 90% predictive values to accurately detect if a swallow was unsafe. This was in the context of improved methodological aspects such as (1) including longer swallow-respiratory sequences, (2) ensuring appropriate minimum CA training, with (3) a structured rating protocol with established CA rating definitions—identified as early CA research flaws [12]. Results from the current study will be further explored below in terms of the study’s primary and secondary outcome measures.

### Primary Outcome: Swallow Safety

Current results show that CA (using swallow-respiratory sequences) demonstrates high validity in detecting if the swallow is safe (sensitivity 85.4%, specificity ≥ 80.3%) across all consistencies combined. These outcomes are congruent with recent CA literature, also using robust methodology with similarly high-very high sensitivities and moderate-high specificities [12, 18, 19, 27]. However, results from the current study differ from early CA research [23, 25, 26] where sensitivities ranged from 62 to 94% with specificities from 56 to 70%, a disparity perhaps due to differences in methodology regarding swallow-respiratory recordings, CA training and rating protocols, as previously reported [12].

In terms of rater reliability, results from the current study demonstrated moderate-good agreement for all consistencies combined. Again, intra rater reliability (83% agreement, Kappa = 0.65) and inter rater reliability (52%, Kf = 0.58) in the current study are comparable to more recent CA research with strong methodology (Kappa = 0.6 – 0.8) versus early research with variable reliability (Kappa = $-$ 0.12 – 0.85). Although, the reliability results from recent CA research present higher rater reliabilities [12, 17] than the present study, variability continues to exist, a phenomenon also seen within instrumental assessments [16]. The current study also identifies higher intra rater than inter rater reliability, a pattern similarly seen within instrumental literature [16, 50–52].

### Secondary Outcomes: Dysphagia and Dysphagia Severity

Regarding the dysphagia question, results from the current study demonstrate high sensitivities to detect if a patient had dysphagia (across consistencies), however a low specificity identified a weakness in this research question, that is, the ability to correctly detect patients who did *not* have dysphagia. One explanation could be that raters may have assumed that all swallows were dysphagic. This assumption, however, is incongruent with results from the primary outcome question, where both high sensitivities and specificities were demonstrated to detect if a swallow was safe. Another possible explanation is the disparity between patient/clinical information that was available to the FEES assessors, that was not available to the CA raters. For all FEES evaluations, the assessors were privy to (1) patient journal/medical chart information, (2) patient diagnosis, (3) patient self-report/interview, (4) immediate observations and general patient status, and (5) oral status and cranial nerve assessment. Furthermore, for FEES assessors, the dysphagia rating was made after a minimum of two bolus presentations of each consistency, whereas the CA rating was made using only one swallow-respiratory sequence per consistency. Perhaps it is this lack of CSE information that contributed to the poorer dysphagia specificity results for the CA condition. Furthermore, it is well known that CA should not be used as a stand-alone tool, as was seen within this study [19–21]. Despite this, it is this stringent research methodology that, although not reflective of clinical practice, is also considered to be a scientific strength of the current study.

Regarding the dysphagia severity rating, although results identified moderate correlation with FEES, this is below the recommended criterion validity of 70% [9]. This moderate validity along with fair-moderate rater reliability (kappa = 0.3–0.49) may be attributed to different factors. Firstly, an influence may be that, again, the CA raters were missing a considerable amount of patient and clinical information from several swallows across several bolus consistencies, as discussed previously. Secondly, the dysphagia severity rating is an ordinal scale, where agreement is always poorer compared with a binary outcome (such as if dysphagia is present, a yes/no question). This has previously been identified as a salient aspect with CA, that swallow-respiratory sound sequences should be (1) considered as part of the holistic CSE and (2) used in a binary yes/no context, and not utilized to grade the severity of dysphagia [12, 18, 19, 27]. Future research continues to be recommended.

### Secondary Outcomes: Effect of Bolus Viscosity

Previous CA research indicates that more accurate results are achieved with thin liquids (IDDSI-0) versus thicker liquids [19, 31], a trend further established in the present study. The current study’s data demonstrated lower aROCs and greater CI ranges for the IDDSI-2 and IDDSI-4 consistencies compared with thin liquids (Table 2). Despite better results with thin liquids compared with thick liquids, the negative predicative values across each and every consistency were > 90% in the current study, highlighting the probability that patients who were rated as *not safe* via CA were truly not safe as per FEES.

However, to further discuss the disparity between thin and thicker liquids outcomes, a possible explanation is that thicker consistencies often require one or more swallows to transit through the pharynx compared with thin liquid swallows [19], and delayed penetration/aspiration may occur after several mouthfuls, rather than immediately, secondary to pharyngeal residue [1, 53]. Consequently, recordings of several swallows for the same thick liquid bolus is recommended—as was presented to FEES raters, though not to CA raters. This adds further strength to the argument that CA should be used as part of the CSE with auscultation occurring over several mouthfuls and/or during an entire meal. The methodology of the present study, although ideal from a research perspective, may not however highlight the full potential of CA accuracy in a clinical setting where CA should be used across a range and number of consistencies and during mealtimes to review fatigue, fluctuations, progression, and swallow-respiratory changes over time [8, 9, 14].

### Clinical Application

This study contributes to the growing body of recent CA research highlighting the value of using swallow-respiratory sound sequences to complement the CSE and follow-up meal-time review. Importantly, this research group strongly advocate the use of EBM principles across all assessments, remembering that the CSE should not replace instrumental evaluations: both are needed for optimal dysphagia diagnostics, trial of interventions and holistic dysphagia management. Results from the current study, however, demonstrate > 90% probability (negative predictive values) that patient swallows that were rated as *not safe* via CA, were in fact truly not safe as per FEES. In clinical settings where instrumental assessment may not be immediately available, adding the swallow-respiratory sound sequences to the CSE may well improve the trained SLP to clinically manage nil per os (NPO) patients and those on thickened liquids for extended periods of time. Optimal dysphagia management should avoid keeping patients unnecessarily NPO for prolonged periods whether waiting for an initial instrumental assessment or reassessment/review for diet upgrade. Furthermore, by combining all assessments and clinical information (i.e., instrumental assessment and CSE + CA), skilled clinicians are well-placed to then better and more regularly review dysphagia management and patient success. This research team strongly advocates for using CA as an adjunct to the CSE/review, and all clinical EBM information attained from both instrumental and CSE + CA assessments, as part of optimal dysphagia management and ongoing review. This holistic dysphagia management underpins person-centered care and facilitates best practice as per the ICF framework.

## Limitations and Future Directions

As with all research, this study has its limitations. For the CA condition, raters were asked to make a clinical decision based only on a single bolus (across a range of fluid consistencies) as opposed to the FEES condition where raters were asked to make the same clinical decisions from several swallows across a range of fluid and food consistencies. Another limitation (also considered a methodological strength) of the current study, was that the CA raters were blinded to patients’ demographics and any other clinical data, whereas FEES assessors were not. Previous studies show that case history, patient report and physical exam play an important role in CSE and in predicting patients at risk of dysphagia [14, 54]. Future research should further investigate using CA as a part of a CSE and routine follow-up, as per Frakking et al. [17]. Another aspect to consider and improve for further research is this study’s sample size. Although 103 swallow-respiratory sounds, from 23 patients, were rated by eight SLPs, this could be revisited in future studies. Finally, application of this study’s results is limited to competent CA-trained clinicians. Previous research has highlighted the poorer validity and variable reliability of CA when clinicians have not received adequate training [12]. The current study was conducted with (1) an inclusion criteria of minimum 1-day CA training, plus (2) provision of one-hour consensus training and (3) a structured rating protocol with established CA rating definitions. All above research limitations and parameters should be considered when extrapolating results to clinical practice.

## Conclusion

Cervical auscultation including swallow-respiratory sound sequences showed high validity across all liquid consistencies (IDDSI 0–4) combined in this study, with FEES being the reference test. Results also demonstrated moderate to very good intra rater reliability and fair to moderate inter rater reliability depending on the research question and bolus consistency. These data support the continued use of CA (with swallow + respiratory sounds) to complement the CSE and ongoing swallow assessment and management. Further research is recommended to investigate the validity and reliability of CA as an adjunct to the CSE, when all patient demographic information, cranial nerve examination, clinical signs and symptoms are provided, as is performed in clinical practice.

## Supplementary Information

Below is the link to the electronic supplementary material.

Supplementary file1 (PDF 306 KB)

Supplementary file2 (PDF 281 KB)

Supplementary file3 (PDF 58 KB)

Supplementary file4 (PDF 286 KB)

## References

[1] Dziewas R (2021). EXPRESS: European stroke organisation and european society for swallowing disorders guideline for the diagnosis and treatment of post-stroke dysphagia. Eur Stroke J.

[2] Attrill S, White S, Murray J, Hammond S, Doeltgen S (2018). Impact of oropharyngeal dysphagia on healthcare cost and length of stay in hospital: a systematic review. BMC Health Serv. Res..

[3] Zuercher P, Moret C, Dziewas R, Schefold J (2019). Dysphagia in the intensive care unit: Epidemiology, mechanisms, and clinical management. Crit Care.

[4] Baijens L (2016). European society for swallowing disorders—European union geriatric medicine society white paper: oropharyngeal dysphagia as a geriatric syndrome. Clin Interv Aging.

[5] American Speech-Language-Hearing Association. Adult dysphagia. 2021. www.asha.org/Practice-Portal/Clinical-Topics/Adult-Dysphagia/. Accessed 22 Sept 2021.

[6] Wirth R (2016). Oropharyngeal dysphagia in older persons – from pathophysiology to adequate intervention: a review and summary of an international expert meeting. Clin Interv Aging.

[7] Jones E, Speyer R, Kertscher B, Denman D, Swan S, Cordier R (2018). Health-related quality of life and oropharyngeal dysphagia: a systematic review. Dysphagia.

[8] Garand K, McCullough G, Crary M, Arvedson J, Dodrill P (2020). Assessment across the life span: the clinical swallow evaluation. Am J Speech-Language Pathol.

[9] Speyer R (2021). White paper by the european society for swallowing disorders: screening and non-instrumental assessment for dysphagia in adults. Dysphagia.

[10] McAllister S, Tedesco H, Kruger S, Ward E, Marsh C, Doeltgen S (2020). Clinical reasoning and hypothesis generation in expert clinical swallowing examinations. Int J Lang Commun Disord.

[11] Sackett D (1997). Evidence-based medicine: how to practice and teach EBM.

[12] Bergström L, Cichero J (2021). Dysphagia management: does structured training improve the validity and reliability of cervical auscultation?. Int J Speech Lang Pathol.

[13] Gutenbrunner C, Nugraha B (2020). Decision-making in evidence-based practice in rehabilitation medicine: proposing a fourth factor. Am J Phys Med Rehabil.

[14] Speech Pathologoy Australia. Dysphagia clinical guidelines. 2012.

[15] Threats T (2007). Use of the ICF in dysphagia management. Semin Speech Lang.

[16] Swan K, Cordier R, Brown T, Speyer R (2019). Psychometric properties of visuoperceptual measures of videofluoroscopic and fibre-endoscopic evaluations of swallowing: a systematic review. Dysphagia..

[17] Frakking T, Chang A, O’Grady K, David M, Weir K (2017). Reliability for detecting oropharyngeal aspiration in children using cervical auscultation. Int J Speech Lang Pathol.

[18] Frakking T, Chang A, O’Grady K, David M, Walker-Smith K, Weir K (2016). The use of cervical auscultation to predict oropharyngeal aspiration in children: a randomized controlled trial. Dysphagia.

[19] Bergström L, Svensson P, Hartelius L (2014). Cervical auscultation as an adjunct to the clinical swallow examination: a comparison with fibre-optic endoscopic evaluation of swallowing. Int J Speech Lang Pathol.

[20] Cichero J, Cichero J, Murdoch B (2006). Clinical assessment, cervical auscultation and pulse oximetry. Dysphagia: foundation, theory and practice.

[21] Lagarde M, Kamalski D, Van Den Engel-Hoek L (2016). The reliability and validity of cervical auscultation in the diagnosis of dysphagia: a systematic review. Clin Rehabil.

[22] Almeida S, Ferlin E, Parente M, Goldani H (2008). Assessment of swallowing sounds by digital cervical auscultation in children. Ann Otol Rhinol Laryngol.

[23] Leslie P, Drinnan M, Finn P, Ford G, Wilson J (2004). Reliability and validity of cervical auscultation: a controlled comparison using videofluoroscopy. Dysphagia.

[24] Leslie P, Drinnan M, Zammit-Maempel I, Coyle J, Ford G, Wilson J (2007). Cervical auscultation synchronized with images from endoscopy swallow evaluations. Dysphagia.

[25] Stroud A, Lawrie B, Wiles C (2002). Inter and intra-rater reliability of cervical auscultation to detect aspiration in patients with dysphagia. Clin Rehabil.

[26] Borr C, Hielscher-Fastabend M, Lücking A (2007). Reliability and validity of cervical auscultation. Dysphagia.

[27] Nozue S (2017). Accuracy of cervical auscultation in detecting the presence of material in the airway. Clin Exp Dent Res.

[28] Santamato A (2009). Acoustic analysis of swallowing sounds: a new technique for assessing dysphagia.

[29] Yagi N (2017). Inappropriate timing of swallow in the respiratory cycle causes breathing-swallowing discoordination. Front Physiol.

[30] Yamashita M (2014). Acoustic characteristics of voluntary expiratory sounds after swallow for detecting dysphagia. J Oral Rehabil.

[31] Frakking T, Chang A, O’Grady K, Yang J, David M, Weir K (2017). Acoustic and perceptual profiles of swallowing sounds in children: normative data for 4–36 months from a cross-sectional study cohort. Dysphagia.

[32] Dozier T, Brodsky M, Michel Y, Walters B, Martin-Harris B (2006). Coordination of swallowing and respiration in normal sequential cup swallows. Laryngoscope.

[33] Martin B, Logemann J, Shaker R, Dodds W (1994). Coordination between respiration and swallowing: respiratory phase relationships and temporal integration. J Appl Physiol.

[34] Hopkins-Rossabi T, Armeson K, Zecker S, Martin-Harris B (2021). Respiratory-swallow coordination and swallowing impairment in head and neck cancer. Head Neck.

[35] Leslie P, Drinnan M, Ford G, Wilson J (2002). Swallow respiration patterns in dysphagic patients following acute stroke. Dysphagia.

[36] Gross R, Atwood C, Ross S, Eichhorn K, Olszewski J, Doyle P (2008). The coordination of breathing and swallowing in Parkinson’s disease. Dysphagia.

[37] Valenzano T, Guida B, Peladeau-Pigeon M, Steele C (2020). Respiratory–swallow coordination in healthy adults during drinking of thin to extremely thick liquids: a research note. J. Speech Lang. Hear. Res..

[38] Gross R, Atwood C, Ross S, Olszewski J, Eichhorn K (2009). The coordination of breathing and swallowing in chronic obstructive pulmonary disease. Am J Respir Crit Care Med.

[39] Bossuyt P et al. Research methods & reporting stard 2015: an updated list of essential items for reporting diagnostic accuracy studies. 2015;5527:1–9. doi: 10.1136/bmj.h5527.7. 10.1136/bmj.h5527 PMC4623764 26511519

[40] Kane S. Post. ClinCalc. (2018). https://clincalc.com/stats/Power.aspx. Accessed on 21 Sept 2021.

[41] Langmore S, Kenneth S, Olsen N (1998). Fiberoptic endoscopic examination of swallowing safety: a new procedure. Dysphagia.

[42] Skeat J, Perry A (2005). Outcome measurement in dysphagia: not so hard to swallow. Dysphagia.

[43] Rosenbek J, Robbins J, Roecker E, Coyle J, Wood J (1996). A penetration-aspiration scale. Dysphagia.

[44] Takahashi K, Groher M, Michi K (1994). Methodology of detecting swallowing sounds. Dysphagia.

[45] WeTransfer (2021). https://wetransfer.com/. Accessed 22 Sept 2021.

[46] Wongpakaran N, Wongpakaran T, Wedding D, Gwet K (2013). A comparison of Cohen’s Kappa and Gwet’s AC1 when calculating inter-rater reliability coefficients: a study conducted with personality disorder samples. BMC Med Res Methodol.

[47] Lange R, Lippa A (2017). Sensitivity and specificity should never be interpreted in isolation without consideration of other clinical utility metrics. Clin Neuropsychol.

[48] Schober P, Schwarte L (2018). Correlation coefficients: appropriate use and interpretation. Anesth Analg.

[49] Altman D (1990). Practical statistics for medical research.

[50] Kelly A, Leslie P, Beale T, Payten C, Drinnan M (2006). Fibreoptic endoscopic evaluation of swallowing and videofluoroscopy: does examination type influence perception of pharyngeal residue severity?. Clin Otolaryngol.

[51] Kelly A, Drinnan M, Leslie P (2007). Assessing penetration and aspiration: how do videofluoroscopy and fiberoptic endoscopic evaluation of swallowing compare?. Laryngoscope.

[52] Pilz W (2016). Observers’ agreement on measurements in fiberoptic endoscopic evaluation of swallowing. Dysphagia..

[53] Leder S, Neubauer P (2016). The Yale pharyngeal residue severity rating scale: an anatomically defined and image-based tool.

[54] Rosenbek J, McCullough G, Wertz R (2004). Is the information about a test important? Applying the methods of evidence-based medicine to the clinical examination of swallowing. J Commun Disord.

