# Supplementary material for: Dysphagia Management and Cervical Auscultation: Reliability and Validity Against FEES
Source: Dysphagia. 2022 Jul 15;38(1):305–14. doi: 10.1007/s00455-022-10468-8 (PMC9873722; doi:10.1007/s00455-022-10468-8)
Supplement: Supplementary file 1 — Supplementary file1 (PDF 306 KB) [file 455_2022_10468_MOESM1_ESM.pdf]

Title: Dysphagia management and cervical auscultation: Reliability and validity against FEES

Journal name: Dysphagia

Online resource 1. Patient characteristics

Diagnosis and medical history	Patient age	Patient admission	FEES	
			Dysphagia rating	PAS score
Amyotrophic Lateral Sclerosis (ALS)	56	Outpatient	3	IDDSI-0 (5 ml)=PAS 1<br>IDDSI-0 (10 ml)=PAS 2<br>IDDSI-2=PAS 1<br>IDDSI-4=PAS 2
Chronic renal failure (Stage 5), dialysis 3/week, ischemic heart disease, previous myocardial infarction. Repeated aspiration pneumonias	80	Outpatient	2-3	IDDSI-0 (5 ml)=PAS 1<br>IDDSI-0 (10 ml)=PAS 2<br>IDDSI-2=PAS 1<br>IDDSI-4=PAS 1
Chronic dysphagia, diagnosed with Schatzki's ring, treated with dilatation.	80	Outpatient	3-4	IDDSI-0 (5 ml)=PAS 2<br>IDDSI-0 (10 ml)=PAS 3<br>IDDSI-2=PAS 1<br>IDDSI-4=PAS 2
Admitted with increased work of breathing, cardiac arrhythmia, constipation and dehydration. Previous pons stroke, back pain, ALS and Graves' disease	71	Inpatient	4	IDDSI-0 (5 ml)=PAS 1<br>IDDSI-0 (10 ml)=PAS 1<br>IDDSI-2 =PAS 1<br>IDDSI-4 =PAS 1
Myasthenia Gravis. Patient describes worsening breathing, non-invasive ventilation (BiPAP) overnight, cough assist.	68	Outpatient	3	IDDSI-0 (5 ml)=PAS 5<br>IDDSI-2=PAS 5<br>IDDSI-4=PAS 5

Dysphagia follow-up after parapharyngeal abscess and tracheostomy. Decannulated at time of assessment.	82	Outpatient	3	IDDSI-0 (5ml)=PAS<br>IDDSI-0 (10ml)=PAS<br>IDDSI-2=PAS<br>IDDSI-4=PAS
Huntington's disease	55	Outpatient	2	IDDSI-0 (5 ml)=PAS 7<br>IDDSI-0 (10 ml)=PAS 7<br>IDDSI-2=PAS 5<br>IDDSI-4=PAS 7-8
Nine-year history of dysphagia following acute aorta aneurysm operation, vocal fold paresis, cricopharyngeal dysfunction. History of prostate cancer, anaemia and kidney failure.	78	Outpatient	2-3	IDDSI-0 (5 ml)=PAS 7<br>IDDSI-0 (10 ml)=PAS X<br>IDDSI-2=PAS 7<br>IDDSI-4=PAS 4
Globus sensation. History of ulcerative colitis.	43	Outpatient	5	IDDSI-0 (5 ml)=PAS 1<br>IDDSI-0 (10 ml)=PAS 1<br>IDDSI-2=PAS 1<br>IDDSI-4=PAS 1
Huntington's disease	65	Outpatient	3	IDDSI-0 (5 ml)=PAS 1<br>IDDSI-2=PAS 1<br>IDDSI-4=PAS 1
Self-reported swallowing difficulties past months, with food getting stuck in the throat. Barium swallow = normal. History of diabetes.	53	Outpatient	5	IDDSI-0 (5 ml)=PAS 1<br>IDDSI-0 (10 ml)=PAS 1<br>IDDSI-2=PAS 1<br>IDDSI-4=PAS 1

Self-reported swallowing difficulties. Globus sensation and vocal fold paresis.	83	Outpatient	5	IDDSI-0 (5 ml)=PAS 1<br>IDDSI-0 (10 ml)=PAS 1<br>IDDSI-2=PAS 1<br>IDDSI-4=PAS 1
Parkinsons Disease (PD) with increasing dysphagia past 3 months. History of cardiac infarct, diabetes myelitis type II.	71	Outpatient	4	IDDSI-0 (5 ml)=PAS 1<br>IDDSI-0 (10 ml)=PAS 1<br>IDDSI-2=PAS 1<br>IDDSI-4=PAS 1
Multiple sclerosis, Barrett's oesophagus, recurrent aspiration related pneumonia	69	Outpatient	4	IDDSI-0=PAS 1<br>IDDSI-2=PAS 1<br>IDDSI-4=PAS 1
Rheumatoid arthritis. Idiopathic dysphagia and voice disorder for over a year.	60	Outpatient	2	IDDSI-0 (5 ml)=PAS 8<br>IDDSI-0 (10 ml)=PAS 8<br>IDDSI-2=PAS 2<br>IDDSI-4=PAS 1
Admitted with acute exacerbation of chronic obstructive pulmonary disease. History of atrial fibrillation, ischemic heart disease, Barrett's oesophagus, kidney disease. Several pnuemonias recent years.	71	Inpatient	3	IDDSI-0 (5 ml)=PAS 7<br>IDDSI-0 (10 ml)=PAS 8<br>IDDSI-2 =PAS 1<br>IDDSI-4 =PAS 1
Self-reported swallowing difficulties<br>History of transient ischaemic attack, mild reflux.	73	Outpatient	5	IDDSI-0 (5 ml)=PAS 1<br>IDDSI-0 (10 ml) =PAS 1<br>IDDSI-2=PAS 1<br>IDDSI-4=PAS 1

Self-reported swallow difficulties and globus sensation. Previous investigations = normal.	52	Outpatient	5	IDDSI-0 (5 ml)=PAS 1<br>IDDSI-0 (10 ml)=PAS 1<br>IDDSI-2=PAS 1<br>IDDSI-4=PAS 1
Self-reported swallowing difficulties<br>History of cardiac infarct, COPD/asthma, smoker.	72	Outpatient	5	IDDSI-0 (5 ml)=PAS 1<br>IDDSI-0 (10 ml)=PAS 1<br>IDDSI-2=PAS 1<br>IDDSI-4=PAS 1
Multiple system atrophy. History of dysphagia symptoms for over 1 year.	72	Outpatient	4	IDDSI-0 (5 ml)=PAS 1<br>IDDSI-0 (10 ml)=PAS 1<br>IDDSI-2=PAS 1<br>IDDSI-4=PAS 1
Parkinson’s disease, cognitive decline and weight loss.	82	Outpatient	2	IDDSI-0 (5 ml)=PAS 4<br>IDDSI-0 (10 ml)=PAS 5<br>IDDSI-2=PAS 3<br>IDDSI-4=PAS 3
Malignant tumour of the neck, radiotherapy completed 3 months prior. Nasogastric tube insitu.	70	Outpatient	2	IDDSI-0 (5 ml)=PAS 5<br>IDDSI-0 (10 ml)=PAS 5<br>IDDSI-2=PAS 1<br>IDDSI-4=PAS 1
Current investigations for suspected Parkinson’s plus disease.	66	Outpatient	4	IDDSI-0 (5 ml)=PAS 1<br>IDDSI-0 (10 ml)=PAS 1<br>IDDSI-2=PAS 1<br>IDDSI-4=PAS 1

Penetration Aspiration Scale (PAS)

Dysphagia ratings adapted from Skeat & Perry. Dysphagia. 2005;20:89-174. dio: 10.1007/s00455-004-0028-z.
