# Supplementary material for: Dysphagia Management and Cervical Auscultation: Reliability and Validity Against FEES
Source: Dysphagia. 2022 Jul 15;38(1):305–14. doi: 10.1007/s00455-022-10468-8 (PMC9873722; doi:10.1007/s00455-022-10468-8)
Supplement: Supplementary file 2 — Supplementary file2 (PDF 281 KB) [file 455_2022_10468_MOESM2_ESM.pdf]

Title: Dysphagia management and cervical auscultation: Reliability and validity against FEES

Journal name: Dysphagia

Online Resource 2. FEES protocol (Part 2, Assessment of swallowing)

Thin liquids (IDDSI-0)
5ml x 2 (via teaspoon)
10ml x 2 (via spoon)
Mouthful x 1 (patient determined mouthful size, via dysphagia 'nosey' cup)
20ml (via several mouthfuls)

Mildly thick liquids (IDDSI-2): Ekströms "nyponsoppa"
5ml x 2 (via teaspoon)
10ml x 2 (via spoon)
(if safe)
10ml x 3 (via spoon)

Extremely thick liquids (IDDSI-4) Ekströms apricot "kräm"
5ml x 2 (via teaspoon)
10ml x 2 (via spoon)
(if safe)
10ml x 3 (via spoon)

Soft biscuit (IDDSI-7)
¼ Göteborg's "Marie" biscuit, dunked in nyponssoppa

Thin liquids (IDDSI-0)
10ml x 2 (via spoon)
and/or mouthful (via dysphagia cup)
