# Supplementary material for: Dysphagia Management and Cervical Auscultation: Reliability and Validity Against FEES
Source: Dysphagia. 2022 Jul 15;38(1):305–14. doi: 10.1007/s00455-022-10468-8 (PMC9873722; doi:10.1007/s00455-022-10468-8)
Supplement: Supplementary file 3 — Supplementary file3 (PDF 58 KB) [file 455_2022_10468_MOESM3_ESM.pdf]

Online Resource 3. Summary of swallow-respiratory sound interpretation

Characteristics	Respiration at rest, pre-swallow	Swallow	Glottal release	Post swallow respirations
Normal	• Tidal respirations (even breathing)	• Crisp, distinct, clear<br>• Quick, coordinated.	• Present	• Expiration post swallow<br>• Return to even breathing (as per pre-swallow)
Abnormal	• Breathing throughout oral phase (liquids)<br>• Audible tongue pumping<br>• Quality of sounds (wet, audible secretions)	• Dissociated<br>• Dull<br>• Constricted<br>• Drawn-out<br>• Choking	• Long apnoea<br>• Absent	• Inspiration post swallow<br>• Increased respiratory rate<br>• Gurgling/bubbling<br>• Crackles, wet, audible secretions (liquid vibrating sounds)<br>• Stridor, wheeze<br>• Coughing, throat-clearing

Summarised from previous research:

Bergström et al., Int. J. Speech. Lang. Pathol. 2014;16(5):517-28. doi: doi: 10.3109/17549507.2013.855259.
Bergström & Cichero. Int. J. Speech. Lang. Pathol. 2021, doi: 10.1080/17549507.2021.1953592.
Frakking et al. Dysphagia. 2016;31:738-748. doi: 10.1007/s00455-016-9727-5.
Cichero & Murdoch (Ed). Dysphagia: foundation, theory and practice. John Wiley & Sons; 2006. pp 178.
Yamashita et al. J. Oral Rehabil. 2014;41:667-674. doi: 10.1111/joor.12184.
