# Supplementary material for: Dysphagia Management and Cervical Auscultation: Reliability and Validity Against FEES
Source: Dysphagia. 2022 Jul 15;38(1):305–14. doi: 10.1007/s00455-022-10468-8 (PMC9873722; doi:10.1007/s00455-022-10468-8)
Supplement: Supplementary file 4 — Supplementary file4 (PDF 286 KB) [file 455_2022_10468_MOESM4_ESM.pdf]

Title: Dysphagia management and cervical auscultation: Reliability and validity against FEES

Journal name: Dysphagia

Online Resource 4. Dysphagia severity – Adapted from AusTOMs

Severity	Descriptor
**0**	**Profound swallowing/feeding impairment**: No swallow initiated. Difficulty in opening mouth. No functional movement of oral and/or pharyngeal structures or musculature. Unable to safely manage any oral intake. Requires full alternative (e.g., PEG, NGT) nutrition.
**1**	**Severe swallowing/feeding impairment**: Swallow initiated but is inconsistent/very delayed/severely disordered. Severe oral and/or pharyngeal impairment with no control of bolus. May sometimes safely take small practice amounts of modified consistencies. Requires mainly alternative (e.g., PEG, NGT) or supplementary nutrition.
**2**	**Moderate/severe swallowing/feeding impairment**: Swallow/initiated but may be inconsistent/ delayed/disordered. Moderate oral and/or pharyngeal impairment with poor control of bolus (e.g., pharyngeal leakage/spillage). Safe on a limited range of consistencies, requires strategies and full supervision to manage oral intake. Some alternative/supplementary feeding (e.g., NGT) required.
**3**	**Moderate swallowing/feeding impairment**: Swallow/initiated consistently but delayed/ abnormal. Moderate oral and/or pharyngeal impairment with limited bolus control (e.g., pooling, residue postswallow, buccal pocketing). Can manage a diet of modified consistencies. Some supervision/strategies may be required. No alternative or supplementary feeding required.
**4**	**Mild swallowing/feeding impairment**: Swallow/initiated consistently with appropriate timing and coordination. Mild oral and/or pharyngeal impairment with mild difficulties in bolus control (e.g., mild pooling or delay). Can manage most consistencies, may require some restrictions in range of consistencies. Independent using strategies, with no supervision required. May eat/drink slowly.
**5**	**No swallowing/feeding impairment**: Swallow/initiated consistently with appropriate timing and coordination and full control of bolus. Can manage a full diet in a timely manner and independently.

Skeat & Perry. Dysphagia. 2005;20:89-174. dio: 10.1007/s00455-004-0028-z.
